# Systematic review of antitumour efficacy and mechanism of metformin activity in prostate cancer models

**DOI:** 10.1002/bco2.187

**Published:** 2022-09-30

**Authors:** Nan Fang Wang, Toni Rose Jue, Jeff Holst, Jennifer H. Gunter

**Affiliations:** ^1^ School of Medical Sciences UNSW Sydney Sydney NSW Australia; ^2^ Prince of Wales Clinical School UNSW Sydney Sydney NSW Australia; ^3^ Australian Prostate Cancer Research Centre‐Queensland, Centre for Genomic and Personalised Health, School of Biomedical Sciences, Faculty of Health, Translational Research Institute Queensland University of Technology (QUT) Brisbane QLD Australia

**Keywords:** mechanism of action, meta‐analysis, metformin, prostate cancer, systematic review

## Abstract

Metformin, the first line pharmacotherapy for type 2 diabetes has demonstrated favourable effects in prostate cancer (PCa) across a range of studies evaluating PCa patient outcomes amongst metformin users. However, a lack of rigorously conducted prospective studies has stalled clinical use in this setting. Despite multiple studies evaluating the mechanisms underpinning antitumour effects of metformin in PCa, to date, no reviews have compared these findings. This systematic review and meta‐analysis consolidates the mechanisms accounting for the antitumour effect of metformin in PCa and evaluates the antitumour efficacy of metformin in preclinical PCa studies. Data were obtained through Medline and EMBASE, extracted by two independent assessors. Risk of bias was assessed using the TOXR tool. Meta‐analysis compared in vivo reductions of PCa tumour volume with metformin. In total, 447 articles were identified with 80 duplicates, and 261 articles excluded based on eligibility criteria. The remaining 106 articles were assessed and 71 excluded, with 35 articles included for systematic review, and eight included for meta‐analysis. The mechanisms of action of metformin regarding tumour growth, viability, migration, invasion, cell metabolism, and activation of signalling cascades are individually discussed. The mechanisms by which metformin inhibits PCa cell growth are multimodal. Metformin regulates expression of multiple proteins/genes to inhibit cellular proliferation, cell cycle progression, and cellular invasion and migration. Published in vivo studies also conclusively demonstrate that metformin inhibits PCa growth. This highlights the potential of metformin to be repurposed as an anticancer agent, warranting further investigation of metformin in the setting of PCa.

## 1 INTRODUCTION

Prostate cancer (PCa), the second most commonly diagnosed cancer amongst men worldwide,^1^ has an incidence rate of almost 60% for men above 65 years.^1^ The global mortality from PCa is estimated to double from 2018 to 2040, reaching 379 005 annual deaths.^1^ These numbers indicate new therapies for advanced metastatic PCa are urgently needed.

The current management for locally advanced or metastatic PCa requires androgen deprivation therapy (ADT)^2^, ^3^; however, ADT has limitations. While approximately 80% of PCa tumours respond to ADT,^4^ 10–20% become refractory within 5 years.^5^ This is known as castrate‐resistant PCa (CRPC) and is defined by increasing PSA levels despite testosterone levels below 50 ng/dl.^3^, ^6^ The median prognosis for CRPC is 2 years.^7^ In addition, ADT has several adverse metabolic and cardiovascular effects^8^ including rapid development of insulin resistance, increased cholesterol, sarcopenic obesity^9^ and an increased risk of diabetes mellitus.^10^ By removing an important regulator of prostate differentiation, ADT also facilitates epithelial to mesenchymal transition (EMT), a key process underlying metastasis and therapeutic resistance.^11^ AR‐signalling inhibitors (ARSIs) such as abiraterone and enzalutamide have been widely adopted for CRPC, in line and in combination with chemotherapies such as docetaxel. However, these drugs only extend survival by a matter of months compared with placebo.^12^ This is partially due to the development of resistance^13^ which highlights the need for new therapeutic strategies that counteract drug resistance and mitigate the significant side effects of the current therapeutics.

Metformin as first‐line pharmacotherapy for type 2 diabetes mellitus (T2DM) patients is used by approximately 120 million people worldwide^14^ with no significant long‐term safety issues identified.^15^ Apart from improving insulin sensitivity, metformin has cardiovascular benefits such as reducing cholesterol levels^16^ and body weight^15^ and may counteract ADT‐induced adverse cardio‐metabolic effects. Various epidemiological studies have shown diabetes patients treated with metformin had reduced risk of cancer, including PCa compared with patients on other antiglycaemic agents such as insulin and secretagogues such as sulphonylureas^17^, ^18^, ^19^, ^20^, ^21^, ^22^ with improved survival outcomes.^23^, ^24^ In contrast, other studies have shown metformin does not significantly reduce the risk of PCa.^22^, ^25^, ^26^ Therefore, multiple systematic reviews and meta‐analyses have been conducted to evaluate the clinical outcome of metformin users, specifically evaluating PCa risk (Table 1) and survival (Table 2). With the exception of three studies,^27^, ^28^, ^29^ most meta‐analyses have indicated metformin does not reduce the risk of PCa (Table 1).^30^, ^31^, ^32^, ^33^, ^34^ However, a significant number of observational studies included in these meta‐analyses were retrospective, which may have significant bias. When only considering prospective studies, there was a slight reduction in PCa risk with metformin (summary relative risk = 0.93; 95% CI: 0.89–0.97).^29^ In terms of clinical outcome, five meta‐analyses conclusively indicated that metformin reduced biochemical recurrence (BCR) of PCa,^28^, ^35^, ^36^, ^37^, ^38^ highlighting the possibility of repurposing this antidiabetic drug as an antitumour agent in PCa (Table 2).

**TABLE 1:** Summary of systematic review/meta‐analyses evaluating metformin effect on PCa risk. Results were obtained from clinical data of diabetic patients

Author	Year	Title	Statistics	Risk of PCa
DeCensi et al.^25^	2010	Metformin and cancer risk in diabetic patients: a systematic review and meta‐analysis	RR: 0.69, 95% CI: 0.61–0.79 *p* value: 0.03	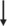
Franciosi et al.^28^	2013	Metformin therapy and risk of cancer in patients with type 2 diabetes: systematic review	OR: 1.18 95% CI: 0.69–2.04 *p* value: 0.545	NS
Zhang et al.^29^	2013	Association of metformin use with cancer incidence and mortality: a meta‐analysis	RR: 0.91 95% CI: 0.80–1.03	NS
Yu et al.^26^	2014	Effect of Metformin on Cancer Risk and Treatment Outcome of Prostate Cancer: A Meta‐Analysis of Epidemiological Observational Studies	OR: 0.91 95% CI: 0.85–0.97 p‐ value: 0.014	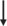
Gandini et al.^27^	2015	Metformin and Cancer Risk and Mortality: A Systematic Review and Meta‐Analysis taking into account Biases and Confounders	SRR: 0.93 95% CI: 0.89–0.97	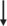
Chen et al.^30^	2018	Metformin, Asian ethnicity and risk of prostate cancer in type 2 diabetes: a systematic review and meta‐analysis	RR: 1.01 95% CI: 0.8–1.28 *p* value: 0.92	NS
Feng et al.^31^	2019	Metformin use and prostate cancer risk: A meta‐analysis of cohort studies	RR: 0.97 95% CI: 0.8–1.16 *p* value: 0.711	NS
Ghiasi et al.^32^	2019	The Relationship Between Prostate Cancer and Metformin Consumption: A Systematic Review and Meta‐analysis Study	OR: 0.89 95% CI: 0.67–1.17	NS

*Note*: NS: No statistically significant relationship between metformin and risk of PCa; RR: relative risk; SRR: summary relative risk; OR: odds ratio, 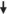: metformin reduces risk of PCa.

**TABLE 2:** Systematic review/meta‐analyses summary of metformin effect on PCa patient outcome

Author	Year		BCR	Overall survival	Cancer specific mortality	Cancer specific survival	All‐cause mortality
Yu et al.^26^	2014	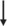	HR: 0.81 95% CI: 0.68–0.98 *p* value: 0.014	‐	‐	‐	NS HR: 0.86 95% CI: 0.64–1.14 *p* value: 0.001
Hwang et al.^33^	2015		RR*:1.20 95% CI*: 1–1.44	‐	NS RR*: 2.27 95% CI*: 0.61–8.38	‐	NS RR*: 1.26 95% CI*: 0.75–2.12
Raval et al.^34^	2015	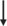	HR: 0.82 95% CI: 0.67–1.01 *p* value: 0.06	‐	NS HR: 0.76 95% CI: 0.43–1.33 *p* value: 0.33.	‐	NS HR: 0.86 95% CI: 0.67–1.1 *p* value: 0.23
Coyle et al.^35^	2016	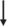	HR 0.83 95% CI: 0.69–1.00	Improved HR: 0.82 95% CI: 0.73–0.93.	‐	Improved HR 0.58 95% CI: 0.37–0.94	‐
Stopsack et al.^36^	2016	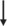	HR: 0.79 95% CI: 0.63–1.00 *p* value = 0.047	Improved HR: 0.88 95% CI: 0.86–0.90 *p* value < 0.001	NS HR 0.76 95% CI: 0.44–1.31 *p* value = 0.33		‐

*Note*: 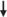: Reduced biochemical recurrence. NS: No significant statistical relationship between metformin use and outcome. ‐: Outcome not investigated in study.

* Statistical results show results of PCa patients with T2DM without the use of metformin.

Multiple in vitro and in vivo studies have investigated the mechanisms accounting for the antitumour effect of metformin in PCa,^39^, ^40^, ^41^ but to date, no reviews have consolidated these findings. Hence, this systematic review and meta‐analysis aims to consolidate the published mechanisms accounting for the antitumour effect of metformin in PCa and evaluate the antitumour efficacy of metformin in preclinical PCa studies.

## 2 METHODS

### 2.1 Search strategy

The search strategy was based on population intervention comparator outcome (PICO) format: What is the cell intrinsic and whole metabolic effects (Outcome) of metformin (Intervention) on prostate cancer (Population)? This systematic review was carried out following the Preferred Reporting Items for Systematic Reviews and Meta‐Analyses (PRISMA) guidelines. N.F.W and J.H.G searched for articles using two search engines: Embase and Medline. Articles published in these search engines up to the date 20 May 2020 were screened. We started with a broad search strategy (Tables S3 and S4), including search terms such as “metformin”, “prostate cancer”, “anti‐proliferative”, “anti‐tumour”, “tumour recurrence”, “metabolism”, “risk”, “morbidity”, and “mortality”. The initial aim was to correlate the antitumour effect of metformin from in vitro studies with clinical outcomes of PCa patients on metformin. Reports containing only clinical studies, without basic or translational components, were excluded as there have been a multitude of systematic reviews evaluating this outcome.^27^, ^30^, ^32^, ^33^, ^35^, ^36^, ^37^, ^38^

### 2.2 Study selection

#### 2.2.1 Inclusion criteria

This review included all studies that investigated mechanisms behind the antitumour effect of metformin in PCa cell lines, mouse models, and in PCa patients.

#### 2.2.2 Exclusion criteria

**FIGURE 1:**
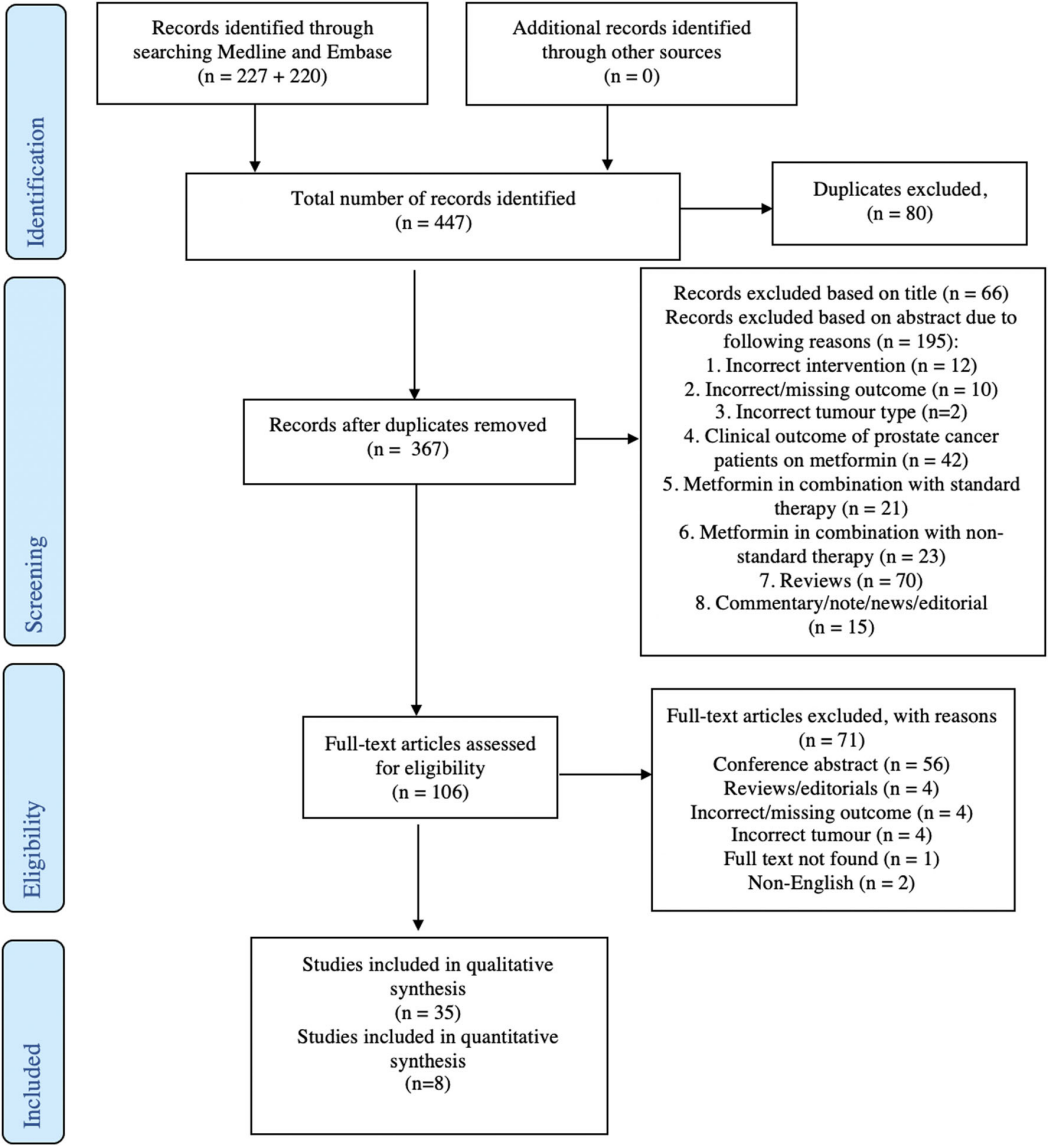
PRISMA flow chart for study inclusion.

This review excluded the following articles (Figure 1):

1. Studies with incorrect intervention.
2. Articles with incorrect/missing outcomes.
3. Studies with incorrect tumour type.
4. Clinical studies of patient's outcome following metformin treatment.
5. Studies investigating metformin effect on standard/non‐standard prostate cancer treatment.
6. Conference abstracts, reviews, notes or editorials.
7. Non‐English language publications.

#### 2.2.3 Data extraction

The authors devised a search strategy to include all possible related synonyms (Tables S3 and S4), identifying 367 articles after duplicates were removed. These articles were screened by both N.F.W and J.H.G based on title, and then abstract and 35 articles were included in this systematic review (Figure 1). Any disagreement in exclusion criteria was resolved via discussion.

Both authors extracted the following data simultaneously from each article: title, author names, year of study, as well as the study objective, outcomes, and methodology. Similar data was collated by both authors.

### 2.3 Risk of bias

To evaluate risk of bias in each article, the Toxicological data Reliability (TOXR) Assessment Tool was used. An 18‐point and 21‐point assessment was evaluated for in vitro and in vivo studies, respectively. The TOXR tool categorizes each article into three different categories: 1, 2, or 3, based on minimum criteria. Category 1 articles are reliable without restrictions; Category 2 articles are reliable with restrictions; Category 3 articles are unreliable and not to be used as a key study. Both authors assessed the risk of bias of each article individually, and discrepancies resolved via discussion.

### 2.4 Statistical analysis

Meta‐analysis was performed on in vivo data to evaluate the effect of metformin treatment on tumour volume. All data were extracted from article texts, tables, and figures, with any estimates made based on the presented data and figures, where tumour volume for control and experimental groups were reported (mean ± standard deviation mm^3^) or could be derived. Derived data included variance estimations based on established statistical methodologies. Statistics were performed on continuous data from 16 experiments from eight studies^40^, ^41^, ^42^, ^43^, ^44^, ^45^, ^46^, ^47^ using random effects model to pool effects sizes for meta‐analysis. Standardized mean difference (Cohen's *d*) was calculated in RevMan software. Heterogeneity was tested using *I* ^2^ statistic. Publication bias was evaluated using funnel plot analysis.

## 3 RESULTS

### 3.1 Study selection and characteristics

A total of 447 articles were identified from Medline and Embase, which included 80 duplicated articles. We screened 367 unique records based on the article title, resulting in 66 articles excluded, which did not match study characteristics. The remaining 301 articles were subjected to screening based on the abstract, with 195 records excluded due to reasons outlined in Figure 1. The full text of the remaining 106 articles was assessed, with a further 71 articles excluded (Figure 1), the majority (56) being conference abstracts. Thus, 35 articles remained for qualitative analysis, and of these, eight studies evaluated the efficacy of metformin in reducing PCa tumour growth in mice models and were included for quantitative analysis (Figure 1).

The 35 articles were published between 2003 and 2018, showing sustained interest in this field. Of the 35 articles included for qualitative analysis, 31 investigated the antitumour effects of metformin using PCa cell lines, 13 articles included mouse models, and five articles included PCa patients. The outcomes of these studies are summarized in Table S5. Out of the 13 articles that included mouse models, eight articles evaluated subcutaneous PCa xenograft tumour volume following metformin administration and utilized similar dosing and experimental duration. The remaining five in vivo articles were excluded as they did not report tumour volume and were instead evaluating metastasis or protein expression.

### 3.2 Qualitative analyses

#### 3.2.1 Risk of bias

The TOXR tool evaluates the reliability of in vitro and in vivo data reporting, assigning weighted scores in the categories of drug identification, in vitro or in vivo model characterization, study design and documented results, with in vivo studies also assessed for plausibility of study design and results. Each criterion within a category is given a score of 1 if the paper contained each relevant detail, or a score of 0 if details were omitted. The 18‐point system from the TOXR tool was used to evaluate all 31 in vitro studies.^48^ Category 1 articles scored 15 or more out of 18, category 2 scored between 11 and 14, and a score of 11 or the inability to meet critical criteria was a category 3 article. Only two of 31 in vitro studies were categorized as category 3 as the critical criterion were not met (Table S6).^42^, ^49^ Caraci et al. did not state the metformin concentration used,^49^ whereas Chen et al.'s study design was inappropriate in achieving the specific aims as it did not evaluate PEDF knockdown or gene inhibition on PCa proliferation, migration, and tumour growth.^42^ Therefore, the findings from these category 3 articles were classified as unreliable. Three articles were assigned to category 2^50^, ^51^, ^52^ as multiple minor criteria were not met including purity of substance,^50^, ^51^, ^52^ experimental incubation temperature and percentage of CO_2_,^50^, ^51^ number of cells seeded,^50^ statistical tests performed,^50^, ^52^ and number of replicates in each experiment.^51^, ^52^ The remaining 26 studies were classified as category 1 and deemed reliable for further analysis (Table S6).^39^, ^40^, ^43^, ^44^, ^46^, ^47^, ^53^, ^54^, ^55^, ^56^, ^57^, ^58^, ^59^, ^60^, ^61^, ^62^, ^63^, ^64^, ^65^, ^66^, ^67^, ^68^, ^69^, ^70^, ^71^

The 21‐point system from the TOXR tool was used to evaluate the 13 in vivo mouse studies. Only one of the 13 studies was classified as category 3 as not all critical criteria were met.^72^ The study design was inappropriate in achieving the aim as mice did not harbour PCa tumours, despite the study hypothesising metformin may interfere with PCa progression.^72^ An additional four studies were classified as category 2^44^, ^45^, ^55^, ^68^ with a score between 13 and 17. Criteria that were not met included purity of substance,^44^, ^45^, ^55^, ^68^ gender of organism,^44^, ^55^, ^68^ age/weight of organism,^44^, ^45^, ^55^ feeding/housing conditions,^45^, ^55^ statistical significance,^44^, ^45^, ^68^ source of metformin,^68^ and vehicle used to dissolve metformin.^68^ The remaining eight studies were classified as category 1, meeting most criteria with a score of 18 or more.^40^, ^41^, ^42^, ^43^, ^46^, ^47^, ^54^, ^62^

Of the 35 articles identified, five articles evaluated the clinical effects of metformin in human PCa patients. Again, the 21 point system from the TOXR tool was used to evaluate each article, with three articles classified as category 3 as critical criteria such as metformin dose,^55^, ^68^ or frequency and duration of exposure to metformin was not disclosed,^55^, ^68^, ^73^ while the remaining two articles were category 1 articles with most/all criteria being met^47^, ^74^ (Table S6).

#### 3.2.2 Type of outcomes

The outcomes most reported for in vitro studies included cell viability, apoptosis, migration, invasion, cell cycle arrest, and protein expression. For in vivo studies, the outcomes included tumour volume, and in a small number of studies, metastatic burden (Table S5).

#### 3.2.3 Cell growth

Major hallmarks of cancer include ability to evade cell death and sustaining proliferative signals.^75^ To evaluate the antitumour effect of metformin, one of the most reported outcomes in the 31 in vitro studies was the effect of metformin on PCa cell viability. The Cell Counting Kit‐8 assay, the 3‐(4,5‐dimethylthiazol‐2‐yl)‐2,5‐diphenyltetrazolium bromide (MTT) assay and related MTS and Alamar blue assays were used to evaluate cell viability. The caveat of these assays is they extrapolate viability from mitochondrial activity and metformin is a known inhibitor of Complex I of the electron transfer chain. Decreased mitochondrial respiration may result in an overestimation of the effects of metformin on cell viability using these approaches.

The extent of inhibition on cell proliferation was dependent on the experimental method (e.g., incubation period, concentration of drug, and cell line used). A range of doses from 0.1–50 mM were tested, with 5 mM the most common concentration, still well above the reported peak‐plasma concentrations obtainable in humans,^76^ with four studies using concentrations in the high micromolar range.^42^, ^45^, ^46^, ^70^ The viability of androgen‐dependent PCa cell lines (LNCaP, VCaP, DuCaP) was evaluated in 15 studies and showed significant inhibition of cell growth (Table S5).^39^, ^40^, ^41^, ^42^, ^46^, ^50^, ^51^, ^52^, ^55^, ^58^, ^59^, ^60^, ^61^, ^64^, ^67^ LNCaP cells treated with 5 mM metformin for 48 h resulted in growth inhibition between 25% and 60% (Table S5).^41^, ^46^, ^50^, ^51^, ^59^, ^64^ There were 20 in vitro studies (*n* = 20) that evaluated viability using androgen‐independent PCa cell lines (DU145, PC‐3, C4‐2, PPC1, LNCaP‐LA, 22Rv1), most commonly PC‐3 and DU145.^39^, ^40^, ^41^, ^42^, ^44^, ^46^, ^47^, ^50^, ^51^, ^52^, ^53^, ^54^, ^55^, ^59^, ^60^, ^61^, ^64^, ^66^, ^67^, ^70^ Metformin reduced cell viability of androgen‐independent PCa cells in 17 out of 20 studies,^39^, ^40^, ^42^, ^44^, ^46^, ^47^, ^50^, ^51^, ^52^, ^53^, ^54^, ^55^, ^59^, ^60^, ^61^, ^66^, ^67^ whereas three studies showed a nonsignificant effect.^41^, ^64^, ^70^ Lastly, six in vitro studies evaluated the outcome of metformin on the cell viability of benign prostatic epithelial cells (PNT1A, RWPE‐1, EP156T, P69),^39^, ^40^, ^41^, ^54^, ^61^, ^70^ which reported no effect in three out of six studies (50%),^54^, ^61^, ^70^ but growth inhibition ranging from 20% to 60% (treated for 24, 72, and 96 h) in the remaining 3.^39^, ^40^, ^41^ In addition, both Wang et al. and Shen et al. showed the combination of metformin and ARSI, bicalutamide resulted in greater inhibition on PCa cell viability compared with either agent alone.^52^, ^59^

#### 3.2.4 Apoptosis

Nine studies evaluated whether reduced viability with metformin was due to apoptosis. Levels of apoptosis were evaluated by flow cytometry and annexin V staining or caspase 3 cleavage.^40^, ^42^, ^44^, ^46^, ^47^, ^52^, ^54^, ^59^, ^70^ All of the studies included androgen‐independent PCa cell lines (C4‐2B, PC‐3, CWR22Rv1, DU145),^40^, ^42^, ^44^, ^46^, ^47^, ^52^, ^54^, ^59^, ^70^ whereas three studies included androgen‐dependent (LNCaP) cell lines,^40^, ^42^, ^59^ and one study included the benign prostatic cell line PNT1A.^70^ The effect of metformin on induction of apoptosis in PCa cells remains relatively controversial, as three out of the nine studies showed that metformin (0.1–10 mM) does not significantly induce apoptosis in androgen‐independent PCa cell lines,^40^, ^46^, ^70^ whereas the remaining six that assessed higher metformin concentrations of 2–30 mM showed increased induction of apoptosis.^42^, ^44^, ^47^, ^52^, ^54^, ^59^ Of the three studies that evaluated metformin and apoptosis of LNCaP cells, one study using 5‐mM metformin showed a nonsignificant effect,^40^ whereas two studies using metformin (0.625–30 mM) showed increased induction of apoptosis following metformin treatment.^42^, ^59^

#### 3.2.5 Cell cycle inhibition

Cell cycle is a tightly regulated process in most functioning cells with multiple checkpoints that trigger cell cycle arrest. However, cancer cells have deregulated cell cycle and increased proliferation.^77^ A total of eight in vitro studies evaluated the effect of metformin on PCa cell cycle progression. The most used method to evaluate cell cycle was flow cytometry.^40^, ^42^, ^47^, ^50^, ^54^, ^58^, ^61^, ^66^ A range of PCa cell lines were used in these studies: Four studies used only androgen‐dependent PCa cell lines (LNCaP, VCaP),^40^, ^50^, ^58^, ^61^ three studies used only androgen‐independent PCa cell lines (C4‐2B, 22Rv1, DU145, PC‐3),^47^, ^54^, ^66^ and one study used both.^42^ The concentration range of metformin ranged from 0.625 to 10 mM, with 5 mM most commonly used. In six of the eight studies, cell cycle progression was inhibited in both androgen‐independent and androgen‐dependent PCa cell lines (LNCaP, VCaP, C4‐2B, 22Rv1, DU145) with an increased percentage of cells in G_0_/G_1_ phase or reduced percentage of cells in S + G_2_/M phase (Table S5).^40^, ^50^, ^54^, ^58^, ^61^, ^66^ For PCa cell lines treated with 5 mM metformin, there was a reduction of 7.1–16.2% of cells in S‐phase following treatment, and an increase of 9.1–24% of cells in G_0_/G_1_ (Table S5).^40^, ^50^, ^61^, ^66^ The variation in percentage reduction could be due to the difference in PCa cell type and/or incubation period. Liu et al. mentioned possible inhibition in PC‐3 and DU145 cell cycle progression by metformin; however, the percentage reduction/increment was neither given in the text nor the supplementary figure.^47^ In contrast, Kato et al. showed 24‐h exposure to metformin (0.625, 2.5, and 10 mM) did not alter cell cycle progression in either PC‐3 or LNCaP cells.^46^

#### 3.2.6 Cellular invasion and migration

The invasive and migratory ability of PCa cells is critical in the development of metastasis.^78^ Thirteen studies evaluated the effect of metformin on PCa cell migration/invasion,^39^, ^41^, ^42^, ^43^, ^46^, ^47^, ^58^, ^61^, ^62^, ^66^, ^68^, ^69^, ^70^ typically using Boyden's chamber or wound healing assays, in androgen‐dependent (LNCaP, VCaP), and androgen‐independent PCa cell lines (PC‐3, DU145, 22Rv1) and benign prostate epithelial cells (PNT1A and RWPE). All five studies that evaluated the effect of metformin on migration and/or invasion of androgen‐dependent PCa cells concluded metformin (0.625–20 mM) reduced cellular migration and/or invasion in androgen‐dependent PCa cell lines.^39^, ^42^, ^47^, ^58^, ^61^ For androgen‐independent PCa cell lines, eight of nine studies that evaluated cellular migration using metformin (1–20 mM)^39^, ^41^, ^42^, ^43^, ^46^, ^66^, ^68^, ^69^ and six out of seven that evaluated cellular invasion using metformin (0.625–20 mM),^42^, ^43^, ^46^, ^62^, ^68^, ^69^ showed similar findings. Therefore, the inhibitory effect of metformin on cellular migration and invasion may be independent of the androgen receptor (AR) signalling pathway.

#### 3.2.7 Protein expression and molecular pathways

**FIGURE 2:**
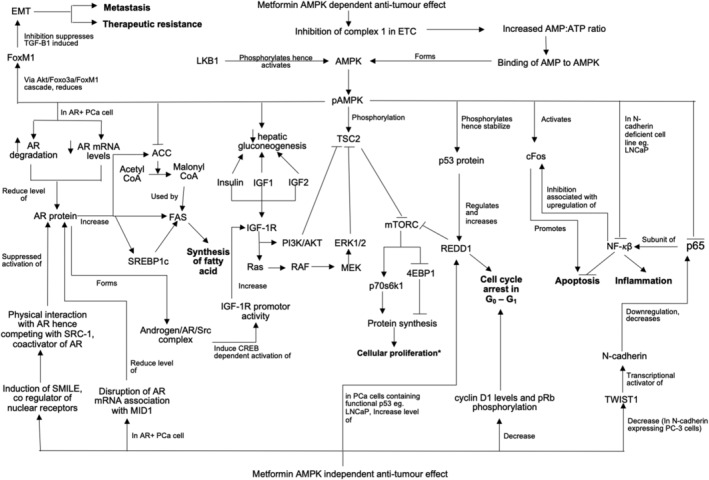
Schematic diagram of AMPK‐dependent and ‐independent anti‐tumour effects of metformin in PCa. The diagram is a summary of findings from the above 37 articles in “metformin molecular anti‐tumour effect”. Abbreviations: AMPK, AMP activated kinase; AR, androgen receptor; ERK, extracellular signal regulated kinase; EMT, epithelial to mesenchymal transition; FAS, fatty acid synthase; LKB1, liver kinase B1; mTORC, mammalian target of rapamycin complex; phosphatidylinositol 3 kinase, PI3K; SREBP1c, sterol regulatory element binding protein 1c; TSC2, tuberous sclerosis complex 2.

**FIGURE 3:**
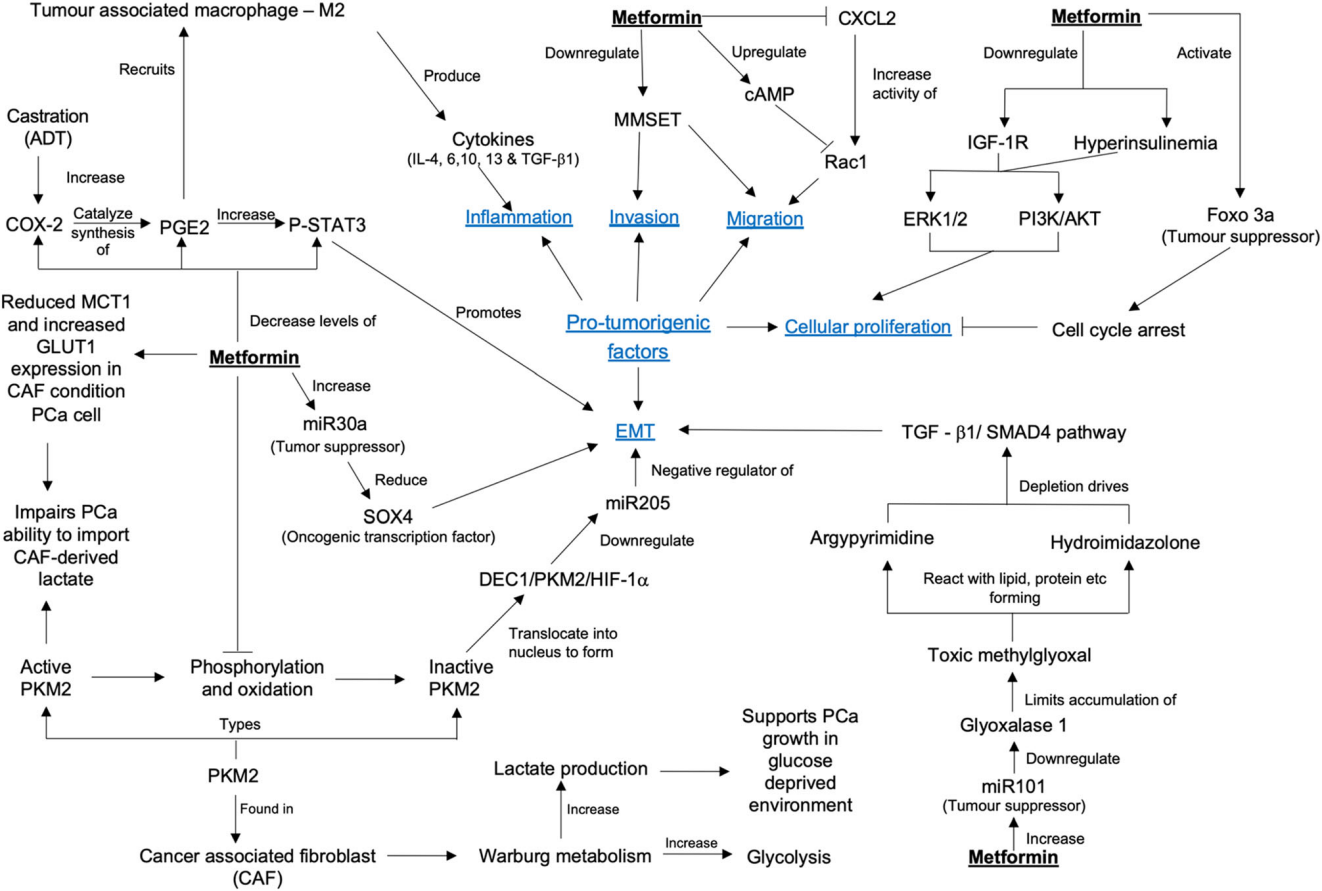
Schematic diagram of other molecular mechanisms accounting for the anti‐tumour effect of metformin in PCa. The role of AMPK in these mechanisms have yet been investigated.

Since Evans et al. first reported in 2005 that metformin use in T2DM patients was associated with a reduced risk of cancer,^79^ multiple preclinical studies have been carried out to delineate its molecular mechanisms. The hallmarks of cancer include the ability of maintaining proliferative signalling, evasion of growth suppressors, evasion of apoptosis, inducing angiogenesis, invasion and metastasis, and lastly allowing for indefinite cellular replication.^75^ Four emerging hallmarks have emerged including reprogramming energy metabolism to allow for rapid cell growth and division, and the ability to evade immune destruction.^75^ From the 31 in vitro studies identified, it appears metformin inhibits a large proportion of these capabilities, summarized in Figures 2 and 3.

Activation of AMP activated kinase (AMPK) is a widely known effect of metformin, which inhibits several metabolic enzymes such as mammalian target of rapamycin (mTOR), and acetyl‐CoA carboxylase (ACC), responsible for protein synthesis and fatty acid synthesis respectively (Figure 2).^40^, ^63^ Both of these processes are commonly elevated in cancer cells.^80^ Therefore, activation of AMPK by metformin could account for one of its antitumour mechanisms, affecting energy triage and controlling cell cycle, biomass accumulation for cell proliferation and apoptosis. Of the 31 in vitro articles, 11 studies evaluated PCa cell lines for AMPK activation by western blot. These studies consistently reported metformin results in increased pAMPK expression while total AMPK levels remained relatively unchanged.^40^, ^44^, ^50^, ^51^, ^53^, ^57^, ^59^, ^60^, ^65^, ^66^, ^70^

Apart from inhibiting ACC and mTORC, the activation of AMPK by metformin has also been associated with the reduction of FoxM1, a transcription factor that regulates EMT, vital for PCa cellular migration.^60^ Knockdown of FoxM1 by shRNA in androgen‐independent DU‐145 cells resulted in reduced cellular migration, decreased expression of mesenchymal markers (vimentin and slug) and increased expression of epithelial markers (E‐cadherin).^60^ These data suggest that metformin may inhibit EMT, a process underlying metastasis and therapeutic resistance, via suppression of FoxM1 in PCa.^60^ Several additional studies have linked metformin with inhibition of EMT in PCa, although whether these pathways are AMPK‐dependent has not been investigated. For instance, metformin treatment resulted in an upregulation of miR30a (a tumour suppressor) and subsequent reduction of SOX4 (oncogenic transcription factor), a known EMT regulator (Figure 3).^61^ Metformin treatment also inhibits the phosphorylation and oxidation of pyruvate kinase M2 (PKM2), thereby preventing the downregulation of miR205, a negative regulator of EMT.^62^ In addition, while ADT is the gold standard treatment for PCa, ADT induces EMT possibly through the upregulation of COX2 and p‐STAT3.^68^ Metformin was shown to reduce expression of COX‐2, PGE2, and p‐STAT3, potentially inhibiting castration‐induced EMT.^68^ Finally, metformin, only at very high concentrations, were shown to increase the tumour suppressor miR‐101 that downregulates expression of glyoxalase 1, which also inhibits EMT (Figure 3).^69^

AR signalling drives PCa growth via regulating cellular proliferation and apoptosis^59^; therefore, reducing AR signalling through ADT is the mainstay treatment for locally advanced PCa.^3^ Metformin reduces AR signalling in both androgen‐independent (LNCaP‐abl) and androgen‐dependent (LNCaP, C4‐2B) PCa cell lines.^39^, ^59^ Shen et al. reported AMPK activation by metformin in LNCaP cells resulted in low AR protein levels via reducing AR mRNA expression and increasing AR protein degradation.^59^ In contrast, Demir et al. found AMPK activation is not required for the reduction of AR levels.^39^ Instead, metformin disrupts AR mRNA association with MID1 translational complex thereby reducing AR protein levels.^39^ In addition, Lee et al. reported that metformin increased small heterodimer partner‐interacting leucine zipper (SMILE), which serves as an AR corepressor, thereby providing a new mechanism to account for the inhibition of AR function in PCa cells, independent of AMPK activation (Figure 2).^51^

Another key molecular pathway implicated in PCa growth is the type 1 insulin‐like growth factor receptor (IGF‐1R) signalling cascade. Inhibition of IGF‐1R using a human monoclonal antibody successfully inhibited both androgen‐independent and ‐dependent prostate tumour growth in vivo.^81^ Activated IGF‐1R results in downstream activation of the phosphatidylinositol 3 kinase/protein kinase‐B (PI3K/Akt) pathway and mitogen‐activated protein kinase (MAPK) pathway thereby increasing cellular proliferation.^82^ One paper reported crosstalk between IGF‐1R and the AR signalling cascade, with androgen stimulation inducing cAMP‐response element‐binding protein (CREB) activation and CREB‐dependent IGF‐1R gene transcription, to upregulate IGF‐1R.^58^ Metformin is capable of not only downregulating AR as mentioned above, but it can also inhibit androgen‐induced cAMP response element (CRE) activity and IGF‐1R gene transcription, thereby reducing IGF‐1‐mediated cell proliferation (Figure 2).^58^ The reduction in IGF‐1R expression is supported by Kato et al. who showed that IGF‐1R mRNA and protein expression decreased following metformin (5 and 10 mM) exposure in PC‐3 cells, and that IGF‐1R knockdown using siRNA inhibited cellular proliferation, invasion, and migration of PC‐3 cells. Daily intraperitoneal injection of metformin in a subcutaneous PC‐3 xenograft model reduced tumour growth and IGF‐1R mRNA expression which further supports the finding that metformin targets IGF‐1R signalling to inhibit PCa growth.^46^

Other AMPK‐independent pathways reported to mediate the antitumour effect of metformin in PCa included upregulation of REDD1^50^ and reduction of cyclin D1 and pRb phosphorylation inducing cell cycle arrest^40^; in N‐cadherin expressing PC‐3 cells, metformin reduced TWIST1 thereby silencing N‐cadherin and decreasing p65 (subunit of NF‐kB) resulting in apoptosis.^44^ However, in N‐cadherin‐deficient LNCaP cells, metformin induced apoptosis via AMPK activation that resulted in inhibition of downstream NF‐kB signalling.^44^, ^83^ Therefore, the metformin antitumour effect may be mediated via different molecular mechanisms depending on the specific PCa cell line (Figure 2).

Other studies have investigated the antitumour mechanism of metformin without evaluating AMPK (Figure 3).^43^, ^47^, ^61^, ^62^, ^66^, ^67^, ^68^, ^69^ In one study, metformin inhibited cellular migration and invasion via downregulation of histone methyltransferase multiple myeloma SET (MMSET),^66^ and inhibition of Rac1.^43^ Metformin has also been reported to activate tumour suppressor Foxo3a resulting in cell cycle arrest^67^ and may affect tumour‐promoting inflammation, as it inhibited infiltration of tumour‐associated macrophages via a reduction in COX2 and PGE_2_ in PCa cells.^47^ Finally, many cancer cells source carbon by increasing glucose uptake and lactate from cancer‐associated fibroblasts (CAFs).^62^ Metformin can reprogram PCa cell metabolism by reducing Glut1 and MCT1 expression in PCa cells, reducing capacity to take up glucose and lactate through these transporters.^62^

### 3.3 Quantitative analysis

#### 3.3.1 In vivo effects of metformin

Metformin was tested in PCa preclinical models. While 13 articles included mouse models, one had an incorrect design and did not include PCa despite their study aims.^72^ In the 12 remaining papers that used metformin in PCa mouse models, three papers used models of spontaneous PCa including the TRAMP model^47^, ^55^ or a Hi‐Myc mouse model (where c‐myc was overexpressed under the control of the probasin promoter)^54^ to assess the effect of metformin on prostatic intraepithelial neoplasia and cancer lesion development. Of the remaining nine studies, four PCa xenograft models were used. Three studies used the AR‐positive cell line LNCaP^40^, ^44^, ^45^ and one study used AR‐positive, androgen insensitive 22RV1 cells.^68^ Most studies used AR‐negative cell lines, PC‐3 cells (six studies) or DU145 (one study).^41^, ^42^, ^43^, ^44^, ^45^, ^46^, ^62^ One study injected PC‐3 cells orthotopically,^43^ and one used injection of PC‐3 cells in the lateral tail vein^62^ in order to assess metformin effect on metastases. The remaining seven studies utilized subcutaneous xenograft models^40^, ^41^, ^42^, ^44^, ^45^, ^46^, ^68^ and measured differences in tumour volume after metformin treatment as their primary outcome.

In the seven subcutaneous models, mice were injected with between 1 and 7 million cells per mouse, although the majority used 1–2.5 million cells. Metformin dose was given daily in drinking water (doses ranging from 100 to 250 mg/kg/day),^40^, ^41^, ^43^, ^44^, ^45^, ^47^, ^54^ gavage (300 mg/kg),^68^ intraperitoneal injection (doses ranging from 20 to 250 mg/kg/day),^40^, ^42^, ^43^, ^44^, ^46^, ^55^ and in one case, PC‐3 cells were pretreated with conditioned media from cancer‐associated fibroblasts grown in media with or without 5 mM metformin, before injection and monitoring of tumour growth without in vivo metformin treatment.^62^

**FIGURE 4:**
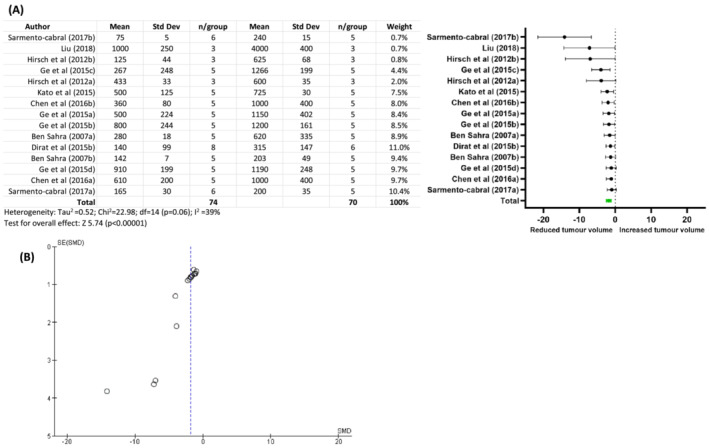
Meta‐analysis of the effect of metformin on growth of subcutaneous prostate cancer tumours. (A) The combined data shows a significant effect of metformin in tumour reduction. Group size and nature of the data comparison results in high degree of heterogeneity. Dirat^43^ was excluded as the study duration was 2 weeks and the remaining studies were 4–6 weeks duration. (B) Funnel plot analysis shows a deviation from the expected mean indicating publication bias with published in vivo studies showing an overwhelmingly inhibitory effect of metformin on tumour growth.

Universally, metformin was reported to reduce tumour initiation, with reduced prostatic intraepithelial neoplasia lesions,^47^, ^54^ reduced metastases consistent with decreased expression of c‐myc and EMT hallmark genes,^54^, ^62^ and reduced tumour growth for both subcutaneous and intraprostatic xenograft PCa models.^40^, ^41^, ^42^, ^43^, ^44^, ^45^, ^46^, ^47^, ^54^ To further evaluate the reported effects of metformin on subcutaneous tumour growth, we performed a meta‐analysis of studies where tumour volume was reported. This limited our meta‐analysis to include eight of the 11 in vivo studies with treatment duration 4–6 weeks (Table S5)^40^, ^41^, ^42^, ^43^, ^44^, ^45^, ^46^, ^47^ and demonstrated a statistically significant drug effect reducing tumour growth (Figure 4A, weighted SMD −1.81, overall effect *Z* = 5.74, *p* < 0.00001), with low study heterogeneity (*I* ^2^ = 39%). Given this strong association, publication bias was assessed by funnel plot (Figure 4B), which demonstrates a shift from the expected mean indicating publication bias for studies showing metformin as an inhibitor of prostate tumour growth in these model systems.

## 4 DISCUSSION

T2DM is associated with an increased risk of a variety of cancers.^20^, ^21^, ^84^ Metformin is the first‐line pharmacotherapy for T2DM, used by at least 120 million people worldwide^14^ and has low toxicity.^15^ Various epidemiological studies have shown that diabetes patients treated with metformin had reduced risk of PCa,^17^, ^18^, ^19^ with improved survival outcomes.^23^, ^24^ However, direct clinical evidence of tissue‐specific activity is lacking, and it has been suggested the effects of metformin on cancer outcomes could be due to the systemic effect of metformin rather than direct antitumour effects. Two meta‐analyses have indicated that metformin is associated with reduced biochemical recurrence as compared with nonusers in T2DM patients with PCa.^28^, ^35^, ^36^, ^37^, ^38^ The underlying mechanism in PCa are multifactorial. Herein, we review and summarize the efficacy and mechanism of action of metformin as an antitumourigenic agent in PCa.

The 15 in vitro studies demonstrate metformin is capable of reducing androgen‐dependent PCa cell growth,^39^, ^40^, ^41^, ^42^, ^46^, ^50^, ^51^, ^52^, ^55^, ^58^, ^59^, ^60^, ^61^, ^64^, ^67^ while 17 studies concluded that metformin inhibited the viability of androgen‐independent PCa cell growth,^39^, ^40^, ^42^, ^44^, ^46^, ^47^, ^50^, ^51^, ^52^, ^53^, ^54^, ^55^, ^59^, ^60^, ^61^, ^66^, ^67^ suggesting that metformin could be combined as anticancer therapy at various stages of PCa. The inhibitory effect of metformin on PCa cellular growth is replicated in vivo where metformin suppresses PCa (androgen‐independent and androgen‐dependent) tumour growth in multiple studies.^40^, ^41^, ^42^, ^43^, ^44^, ^45^, ^46^, ^47^

The molecular mechanisms by which metformin inhibits PCa growth are highly complex (Figures 2 and 3) and multimodal. AMPK is a master regulator of homeostasis that has a controversial role. While AMPK was identified as a tumour suppressor initially,^85^, ^86^, ^87^, ^88^ multiple studies have also reported a protumourigenic role.^89^, ^90^, ^91^ Several AMPK‐dependent^44^, ^59^, ^60^ and AMPK‐independent pathways^39^, ^40^, ^44^, ^51^ for metformin activity have been identified in this study; however, not all studies evaluated whether AMPK was involved in the antitumour effect of metformin.^43^, ^47^, ^61^, ^62^, ^66^, ^67^, ^68^, ^69^ Regardless of the mechanisms, most of the studies included in this review showed an antitumour role of metformin. This is mediated in a variety of ways including inhibition of cell cycle progression,^40^, ^50^, ^54^, ^58^, ^61^, ^66^ induction of apoptosis,^42^, ^44^, ^47^, ^52^, ^54^, ^59^ inhibition of EMT,^60^, ^61^, ^62^, ^68^, ^69^ blocking cellular invasion and/or migration,^39^, ^41^, ^42^, ^43^, ^46^, ^66^, ^68^, ^69^ reducing tumour‐enabling inflammation,^47^ or changing metabolism.^62^, ^63^ Mechanistically, the two most activated oncogenic signalling cascades are the AR and PI3K/AKT signalling pathways. These two pathways cross regulate each other such that the inhibition of one pathway activates the other to allow PCa survival. Hence, combined pharmacological inhibition of these two pathways resulted in complete regression in PCa xenografts.^92^ Metformin downregulates IGF‐1R expression hence reducing downstream activation of AKT,^46^, ^58^ whereas ARSIs or ADT reduces AR signalling,^3^ therefore the combination of both could potentially inhibit the cross regulation of the two main oncogenic signalling pathways driving PCa growth.

ADT, with sequential addition of ARSI are the mainstay treatment for advanced PCa.^3^, ^12^ However, it has been shown that castration or ARSIs could induce EMT,^13^, ^68^ a process underlying metastasis and therapeutic resistance.^68^ Metformin inhibited castration‐induced EMT and reversed ARSI (e.g., enzalutamide) resistance, thereby suggesting that the combination of metformin with ADT or ARSI could be a possible therapeutic option for advanced PCa.^13^, ^68^ Moreover, in vitro PCa studies that evaluated the combination treatment of an ARSI (bicalutamide) with metformin showed greater growth inhibition in the combination treatment as compared with either agent used alone.^52^, ^59^, ^93^ Liu et al. evaluated the effect of an ARSI (enzalutamide) with metformin in PCa xenografts in vivo, showing that the combination treatment also had a greater inhibition on tumour growth than metformin alone, although in that study enzalutamide on its own had no effect.^13^ These data support the idea that metformin in combination with ADT or ARSI may be a potential therapeutic option for PCa patients. In a recent randomized controlled trial (RCTs) of patients with advanced PCa, the addition of metformin to standard of care resulted in improved cancer‐free survival for both patients with high risk localized disease and patients with metastatic low tumour volume disease.^94^

Metformin also appears to have a role in secondary prevention of PCa. Five meta‐analyses conclusively indicated that metformin reduced biochemical recurrence (BCR) of PCa,^28^, ^35^, ^36^, ^37^, ^38^ highlighting the possibility of repurposing this antidiabetic drug as an antitumour agent in PCa of various stages (Table 2). Until recently, no RCT have been done to ascertain the effect of metformin in low risk PCa.^95^ However, given the promising data seen in preclinical and clinical studies in the last decade, the Metformin Active Surveillance Trial (MAST) has been set up to evaluate the role of metformin in reducing progression amongst men on expectant management for low risk PCa. This is a phase III randomized, double‐blinded, placebo‐controlled trial that is ongoing and estimated to be completed by 2024.^96^

### 4.1 Strength and limitations

The strength of this study lies in the comprehensive search strategy, which included as many articles as possible, therefore providing a holistic overview of the mechanisms and antitumour effect of metformin in PCa. Moreover, all included articles (*n* = 35) have been assessed for data reliability, with the majority being classified as category 1 articles; hence, the findings are reliable.

Nonetheless, this study has several limitations. First, for the in vivo findings, all but one article showed inhibition on PCa tumour growth, suggesting the following possibilities—(1) metformin indeed is potent in terms of inhibiting PCa tumour growth in mice models; or (2) nonsignificant results are rarely published in this field, such that most of the published findings are skewed towards the positive side. Secondly, most of the studies published identified a novel mechanism, and these findings have not yet been verified by other in vitro or in vivo studies. Thirdly, the concentration for metformin used for the in vitro studies were much higher than plasma concentration of metformin in diabetic patients.^76^ Most of the experiments used a concentration of 1–50 mM (Table S5), whereas the plasma concentration of patients taking metformin varies between 0.3 and 1.3 mg/L and maximum obtainable concentration of 3 mg/L, which equates to 1–20 μM.^76^, ^97^ Hence, whether there is an antitumour effect seen at therapeutically relevant plasma concentrations of metformin has not been determined. However, it is important to note that metformin accumulates across the mitochondrial matrix to target the prostate tumour energetics directly. The concentration of metformin in prostate tissue has been shown to be approximately two to 32 times higher than that in serum of PCa patients.^74^

In addition, in vitro studies are generally not conducted to take into consideration the pharmacokinetics of metformin, where peak plasma concentrations occur 2 h post‐dose and the elimination half‐life in patients with good renal function is approximately 5 h.^76^ In addition, all in vitro experiments reviewed were conducted over experimental time frames of 24–96 h and at concentrations an order of magnitude higher than found in plasma. This discrepancy is a source of controversy in the field. With limited evidence of tissue‐specific concentrations,^76^ particularly over sustained treatment periods,^98^ even with adequate controls, differences in effective metformin dose are difficult to compare and may be a function of cancer cell type and predominant metabolic (glycolytic or oxidative) and glucose addiction.^99^ Future in vitro studies could better recapitulate therapeutic plasma concentrations of metformin at steady state observed in T2DM patients. This may remove half‐life as a confounding factor until further studies of intratumoural concentrations of metformin can be ascertained over sustained treatment times.^98^

### 4.2 Conclusion

In conclusion, this systematic review summarizes the in vitro antitumour effects of metformin in PCa and meta‐analysis on the inhibition of PCa growth in mouse models by administration of metformin. The findings from preclinical studies support the idea that metformin could be repurposed as an anticancer therapeutic option in PCa either alone or in conjunction with current therapies (ADT or ARSIs). This is based on the following factors: (1) The ability to inhibit PCa growth in vitro and in vivo, (2) reduce ADT‐induced cardiovascular adverse effects, and (3) reversing castration‐ or treatment‐induced EMT.

### 4.3 Future directions

Given the meta‐analysis revealed publication bias in the in vivo studies, more preclinical data may be needed to ascertain the antitumour effect of metformin in PCa mouse models. In addition, the majority of the clinical studies that evaluated the clinical outcome of PCa patients who are on metformin are retrospective in nature, which has inherent biases,^29^ such as selection bias, interviewer bias or recall bias. Thus, more long‐term RCTs are needed to ascertain whether the inhibitory effect of metformin on PCa growth in preclinical studies are applicable to PCa patients. This is a rapidly evolving field, with the first RCT being published last year showing that metformin combination with ADT improves the outcome for patients with advanced PCa.^94^ While the MAST (a randomized, double‐blinded, placebo‐controlled trial), which looks at the role of metformin in early stage PCa, is currently ongoing and will be completed in 2024.^96^

## CONFLICTS OF INTEREST

All authors have completed the ICMJE uniform disclosure form and declare no support from any organization for the submitted work, no financial relationships with any organizations that might have an interest in the submitted work in the previous 3 years, and no other relationships or activities that could appear to have influenced the submitted work.

## AUTHOR CONTRIBUTION

NFW and JH conceptualized the review, NFW performed the literature search, and NFW and JHG undertook independent review, quality scoring, and meta‐analysis. NFW, TRJ, JH, and JHG wrote the article, and JH and JHG are guarantors of the manuscript. The corresponding author attests that all listed authors meet authorship criteria and that no others meeting the criteria have been omitted.

## TRANSPARENCY STATEMENT

The guarantors affirm that our manuscript is an honest, accurate, and transparent account of the study being reported; that no important aspects of the study have been omitted; and that any discrepancies from the study as originally planned (and, if relevant, registered) have been explained. This review was not registered. All protocols are presented with this submission.

## PATIENT AND PUBLIC INVOLVEMENT

Patient and public involvement was not engaged for this study.

## Supporting information


**Table S3:** **Medline search strategy.** Articles till 15 May 2020

**Table S4:** **Embase search strategy.** Articles included from 2004 till 20 May 2020.

**Table S5.** Supporting Information

**Table S6.** Supporting Information

## References

[1] Rawla P . Epidemiology of prostate Cancer. World J Oncol. 2019;10(2):63–89. 10.14740/wjon1191 31068988 PMC6497009

[2] Ceder Y , Bjartell A , Culig Z , Rubin MA , Tomlins S , Visakorpi T . The molecular evolution of castration‐resistant prostate Cancer. Eur Urol Focus. 2016;2(5):506–13. 10.1016/j.euf.2016.11.012 28723516

[3] Crawford ED , Heidenreich A , Lawrentschuk N , Tombal B , Pompeo ACL , Mendoza‐Valdes A , et al. Androgen‐targeted therapy in men with prostate cancer: Evolving practice and future considerations. Prostate Cancer Prostatic Dis. 2019;22(1):24–38. 10.1038/s41391-018-0079-0 30131604 PMC6370592

[4] Salonen AJ , Viitanen J , Lundstedt S , Ala‐Opas M , Taari K , Tammela TLJ , et al. Finnish multicenter study comparing intermittent to continuous androgen deprivation for advanced prostate Cancer: Interim analysis of prognostic markers affecting initial response to androgen deprivation. J Urol. 2008;180(3):915–20. 10.1016/j.juro.2008.05.009 18635219

[5] Kirby M , Hirst C , Crawford ED . Characterising the castration‐resistant prostate cancer population: A systematic review. Int J Clin Pract. 2011;65(11):1180–92. 10.1111/j.1742-1241.2011.02799.x 21995694

[6] Bellmunt J , Oh WK . Castration‐resistant prostate cancer: New science and therapeutic prospects. Ther Adv Med Oncol. 2010;2(3):189–207. 10.1177/1758834009359769 21789134 PMC3126017

[7] Andersen RJ , Mawji NR , Wang J , Wang G , Haile S , Myung JK , et al. Regression of castrate‐recurrent prostate Cancer by a small‐molecule inhibitor of the amino‐terminus domain of the androgen receptor. Cancer Cell. 2010;17(6):535–46. 10.1016/j.ccr.2010.04.027 20541699

[8] Gupta D , Lee Chuy K , Yang JC , Bates M , Lombardo M , Steingart RM . Cardiovascular and metabolic effects of androgen‐deprivation therapy for prostate Cancer. J Oncol Pract. 2018;14(10):580–7. 10.1200/JOP.18.00178 30312560

[9] Smith MR , Finkelstein JS , Mcgovern FJ , Zietman AL , Fallon MA , Schoenfeld DA , et al. Changes in body composition during androgen deprivation therapy for prostate Cancer. J Clin Endocrinol Metabol. 2002;87(2):599–603. 10.1210/jcem.87.2.8299 11836291

[10] Keating NL , O'malley AJ , Smith MR . Diabetes and cardiovascular disease during androgen deprivation therapy for prostate cancer. J Clin Oncol. 2006;24(27):4448–56. 10.1200/JCO.2006.06.2497 16983113

[11] Sun Y , Wang BE , Leong KG , Yue P , Li L , Jhunjhunwala S , et al. Androgen Deprivation Causes Epithelial–Mesenchymal Transition in the Prostate: Implications for Androgen‐Deprivation Therapy. Cancer Res. 2012;72(2):527–36. 10.1158/0008-5472.CAN-11-3004 22108827

[12] Scher HI , Fizazi K , Saad F , Taplin ME , Sternberg CN , Miller K , et al. Increased survival with enzalutamide in prostate cancer after chemotherapy. N Engl J Med. 2012;367(13):1187–97. 10.1056/NEJMoa1207506 22894553

[13] Liu Q , Tong D , Liu G , Xu J , Do K , Geary K , et al. Metformin reverses prostate cancer resistance to enzalutamide by targeting TGF‐beta1/STAT3 axis‐regulated EMT. Cell Death Dis. 2017;8(8):e3007. 10.1038/cddis.2017.417 28837141 PMC5596596

[14] Viollet B , Guigas B , Garcia NS , Leclerc J , Foretz M , Andreelli F . Cellular and molecular mechanisms of metformin: An overview. Clin Sci (Lond). 2012;122(6):253–70. 10.1042/CS20110386 22117616 PMC3398862

[15] Diabetes prevention program research, G. long‐term safety, tolerability, and weight loss associated with metformin in the diabetes prevention program outcomes study. Diabetes Care. 2012;35(4):731–7. 10.2337/dc11-1299 22442396 PMC3308305

[16] Xu T , Brandmaier S , Messias AC , Herder C , Draisma HHM , Demirkan A , et al. Effects of metformin on metabolite profiles and LDL cholesterol in patients with type 2 diabetes. Diabetes Care. 2015;38(10):1858–67. 10.2337/dc15-0658 26251408

[17] Tseng CH . Metformin significantly reduces incident prostate cancer risk in Taiwanese men with type 2 diabetes mellitus. Eur J Cancer. 2014;50:2831–7. 25201464 10.1016/j.ejca.2014.08.007

[18] Kuo YJ , Sung FC , Hsieh PF , Chang HP , Wu KL , Wu HC . Metformin reduces prostate cancer risk among men with benign prostatic hyperplasia: A nationwide population‐based cohort study. Cancer Med. 2019;8(5):2514–23. 10.1002/cam4.2025 30968600 PMC6536940

[19] Preston MA , Riis AH , Ehrenstein V , Breau RH , Batista JL , Olumi AF , et al. Metformin use and prostate cancer risk. Eur Urol. 2014;66(6):1012–20. 10.1016/j.eururo.2014.04.027 24857538

[20] Mekuria AA‐O , Ayele YA‐O , Tola AA‐O , Mishore KA‐O . Monotherapy with metformin versus sulfonylureas and risk of Cancer in type 2 diabetic patients. A Systematic Review and Meta‐Analysis. 2019;2019:1–8. 10.1155/2019/7676909 PMC6885827 31828167

[21] Thakkar B. , Aronis Kn Fau‐Vamvini MT , Vamvini Mt Fau‐Shields K , Shields K Fau‐Mantzoros CS , Mantzoros, C.S. Metformin and sulfonylureas in relation to cancer risk in type II diabetes patients: a meta‐analysis using primary data of published studies. (2013). 10.1016/j.metabol.2013.01.014 23419783

[22] Currie CJ , Poole CD , Gale EA . The influence of glucose‐lowering therapies on cancer risk in type 2 diabetes. Diabetologia. 2009;52(9):1766–77. 10.1007/s00125-009-1440-6 19572116

[23] Spratt DE , Zhang C , Zumsteg ZS , Pei X , Zhang Z , Zelefsky MJ . Metformin and prostate cancer: Reduced development of castration‐resistant disease and prostate cancer mortality. Eur Urol. 2013;63(4):709–16. 10.1016/j.eururo.2012.12.004 23287698 PMC4034673

[24] He XX , Tu SM , Lee MH , Yeung SCJ . Thiazolidinediones and metformin associated with improved survival of diabetic prostate cancer patients. Ann Oncol. 2011;22(12):2640–5. 10.1093/annonc/mdr020 21415239 PMC3221513

[25] Home PD , Kahn SE , Jones NP , Noronha D , Beck‐Nielsen H , Viberti G , et al. Experience of malignancies with oral glucose‐lowering drugs in the randomised controlled ADOPT (A Diabetes Outcome Progression Trial) and RECORD (Rosiglitazone Evaluated for Cardiovascular Outcomes and Regulation of Glycaemia in Diabetes). Clin Trials. 2010;53(9):1838–45. 10.1007/s00125-010-1804-y PMC2910882 20532476

[26] Hsieh MC , Lee TC , Cheng SM , Tu ST , Yen MH , Tseng CH . The influence of type 2 diabetes and glucose‐lowering therapies on cancer risk in the Taiwanese. (2012), 2012, 1, 6, 10.1155/2012/413782 PMC3374948 22719752

[27] Decensi A , Puntoni M , Goodwin P , Cazzaniga M , Gennari A , Bonanni B , et al. Metformin and cancer risk in diabetic patients: A systematic review and meta‐analysis. Cancer Prev Res (Phila pa). 2010;3(11):1451–61. 10.1158/1940-6207.CAPR-10-0157 20947488

[28] Yu H , Yin L , Jiang X , Sun X , Wu J , Tian H , et al. Effect of metformin on cancer risk and treatment outcome of prostate cancer: A meta‐analysis of epidemiological observational studies. PLoS ONE [Electronic Resource]. 2014;9(12):e116327. 10.1371/journal.pone.0116327 25545701 PMC4278883

[29] Gandini S , Puntoni M , Heckman‐Stoddard BM , Dunn BK , Ford L , Decensi A , et al. Metformin and cancer risk and mortality: A systematic review and meta‐analysis taking into account biases and confounders. Cancer Prev Res (Phila). 2014;7(9):867–85. 10.1158/1940-6207.CAPR-13-0424 24985407 PMC4154969

[30] Franciosi M , Lucisano G , Lapice E , Strippoli GFM , Pellegrini F , Nicolucci A . Metformin therapy and risk of cancer in patients with type 2 diabetes: Systematic review. PLoS ONE. 2013;8(8):e71583. 10.1371/journal.pone.0071583 23936520 PMC3732236

[31] Zhang P , Li H , Tan X , Chen L , Wang S . Association of metformin use with cancer incidence and mortality: A meta‐analysis. Cancer Epidemiol. 2013;37(3):207–18. 10.1016/j.canep.2012.12.009 23352629

[32] Chen CB , Eskin M , Esurich DT , Majumdar SR , Johnson JA . Metformin, Asian ethnicity and risk of prostate cancer in type 2 diabetes: A systematic review and meta‐analysis. BMC Cancer. 2018;18(1):65. 10.1186/s12885-017-3934-9 29320995 PMC5763543

[33] Feng Z , Zhou X , Liu N , Wang J , Chen X , Xu X . Metformin use and prostate cancer risk: A meta‐analysis of cohort studies. Medicine (Baltimore). 2019;98(12):e14955. 10.1097/MD.0000000000014955 30896668 PMC6709307

[34] Ghiasi B , Sarokhani D , Najafi F , Motedayen M , Dehkordi AH . The relationship between prostate Cancer and metformin consumption: A systematic review and Meta‐analysis study. Curr Pharm des. 2019;25(9):1021–9. 10.2174/1381612825666190215123759 30767734

[35] Hwang IC , Park SM , Shin D , Ahn HY , Rieken M , Shariat SF . Metformin association with lower prostate cancer recurrence in type 2 diabetes: A systematic review and meta‐analysis. Asian Pac J Cancer Prev: Apjcp. 2015;16(2):595–600. 10.7314/APJCP.2015.16.2.595 25684493

[36] Raval AD , Thakker D , Vyas A , Salkini M , Madhavan S , Sambamoorthi U . Impact of metformin on clinical outcomes among men with prostate cancer: A systematic review and meta‐analysis. Prostate Cancer Prostatic Dis. 2015;18(2):110–21. 10.1038/pcan.2014.52 25667109 PMC4904224

[37] Coyle C , Cafferty FH , Vale C , Langley RE . Metformin as an adjuvant treatment for cancer: A systematic review and meta‐analysis. Ann Oncol. 2016;27(12):2184–95. 10.1093/annonc/mdw410 27681864 PMC5178140

[38] Stopsack KH , Ziehr DR , Rider JR , Giovannucci EL . Metformin and prostate cancer mortality: A meta‐analysis. Cancer Causes Control. 2016;27(1):105–13. 10.1007/s10552-015-0687-0 26537119

[39] Demir U , Koehler A , Schneider R , Schweiger S , Klocker H . Metformin anti‐tumor effect via disruption of the MID1 translational regulator complex and AR downregulation in prostate cancer cells. BMC Cancer. 2014;14(1):52. 10.1186/1471-2407-14-52 24484909 PMC3929757

[40] Sahra IB , Laurent K , Loubat A , Giorgetti‐Peraldi S , Colosetti P , Auberger P , et al. The antidiabetic drug metformin exerts an antitumoral effect in vitro and in vivo through a decrease of cyclin D1 level. Oncogene. 2008;27(25):3576–86. 10.1038/sj.onc.1211024 18212742

[41] Sarmento‐Cabral A , L‐López F , Gahete MD , Castaño JP , Luque RM . Metformin reduces prostate tumor growth, in a diet‐dependent manner, by modulating multiple signaling pathways. Mol Cancer Res: MCR. 2017;15(7):862–74. 10.1158/1541-7786.MCR-16-0493 28385910

[42] Chen X , Li C , He T , Mao J , Li C , Lyu J , et al. Metformin inhibits prostate cancer cell proliferation, migration, and tumor growth through upregulation of PEDF expression. Cancer Biol Ther. 2016;17(5):507–14. 10.1080/15384047.2016.1156273 26987032 PMC4910909

[43] Dirat B , Ader I , Golzio M , Massa F , Mettouchi A , Laurent K , et al. Inhibition of the GTPase Rac1 mediates the antimigratory effects of metformin in prostate cancer cells. Mol Cancer Ther. 2015;14(2):586–96. 10.1158/1535-7163.MCT-14-0102 25527635

[44] Ge R , Wang Z , Wu S , Zhuo Y , Otsetov AG , Cai C , et al. Metformin represses cancer cells via alternate pathways in N‐cadherin expressing vs. N‐Cadherin Deficient Cells. Oncotarget. 2015;6(30):28973–87. 10.18632/oncotarget.5023 26359363 PMC4745705

[45] Hirsch HA , Iliopoulos D , Struhl K . Metformin inhibits the inflammatory response associated with cellular transformation and cancer stem cell growth. Proc Natl Acad Sci U S A. 2013;110(3):972–7. 10.1073/pnas.1221055110 23277563 PMC3549132

[46] Kato H , Sekine Y , Furuya Y , Miyazawa Y , Koike H , Suzuki K . Metformin inhibits the proliferation of human prostate cancer PC‐3 cells via the downregulation of insulin‐like growth factor 1 receptor. Biochem Biophys Res Commun. 2015;461(1):115–21. 10.1016/j.bbrc.2015.03.178 25862373

[47] Liu Q , Tong D , Liu G , Gao J , Wang LA , Xu J , et al. Metformin inhibits prostate Cancer progression by targeting tumor‐associated inflammatory infiltration. Clin Cancer Res. 2018;24(22):5622–34. 10.1158/1078-0432.CCR-18-0420 30012567

[48] European Commission ToxRTool (https://ec.europa.eu/jrc/en/scientific-tool/toxrtool-toxicological-data-reliability-assessment-tool 2009).

[49] Caraci F , Chisari M , Frasca G , Chiechio S , Salomone S , Pinto A , et al. Effects of phenformin on the proliferation of human tumor cell lines. Life Sci. 2003;74(5):643–50. 10.1016/j.lfs.2003.07.015 14623034

[50] Ben Sahra I , Regazzetti C , Robert G , Laurent K , Le Marchand‐Brustel Y , Auberger P , et al. Metformin, independent of AMPK, induces mTOR inhibition and cell‐cycle arrest through REDD1. Cancer Res. 2011;71(13):4366–72. 10.1158/0008-5472.CAN-10-1769 21540236

[51] Lee SY , Song CH , Xie YB , Jung C , Choi HS , Lee K . SMILE upregulated by metformin inhibits the function of androgen receptor in prostate cancer cells. Cancer Lett. 2014;354(2):390–7. 10.1016/j.canlet.2014.09.001 25199764

[52] Wang Y , Liu G , Tong D , Parmar H , Hasenmayer D , Yuan W , et al. Metformin represses androgen‐dependent and androgen‐independent prostate cancers by targeting androgen receptor. Prostate. 2015;75(11):1187–96. 10.1002/pros.23000 25894097

[53] Zakikhani M , Dowling RJ , Sonenberg N , Pollak MN . The effects of adiponectin and metformin on prostate and colon neoplasia involve activation of AMP‐activated protein kinase. Cancer Prev Res (Phila pa). 2008;1(5):369–75. 10.1158/1940-6207.CAPR-08-0081 19138981

[54] Akinyeke T , Matsumura S , Wang X , Wu Y , Schalfer ED , Saxena A , et al. Metformin targets c‐MYC oncogene to prevent prostate cancer. Carcinogenesis. 2013;34(12):2823–32. 10.1093/carcin/bgt307 24130167 PMC3845895

[55] Fendt SM , Bell EL , Keibler MA , Davidson SM , Wirth GJ , Fiske B , et al. Metformin decreases glucose oxidation and increases the dependency of prostate cancer cells on reductive glutamine metabolism. Cancer Res. 2013;73(14):4429–38. 10.1158/0008-5472.CAN-13-0080 23687346 PMC3930683

[56] Moiseeva O , Deschênes‐Simard X , St‐Germain E , Igelmann S , Huot G , Cadar AE , et al. Metformin inhibits the senescence‐associated secretory phenotype by interfering with IKK/NF‐kappaB activation. Aging Cell. 2013;12(3):489–98. 10.1111/acel.12075 23521863

[57] Chou CC , Lee KH , Lai IL , Wang D , Mo X , Kulp SK , et al. AMPK reverses the mesenchymal phenotype of cancer cells by targeting the Akt‐MDM2‐Foxo3a signaling axis. Cancer Res. 2014;74(17):4783–95. 10.1158/0008-5472.CAN-14-0135 24994714 PMC4155002

[58] Malaguarnera R , Sacco A , Morcavallo A , Squatrito S , Migliaccio A , Morrione A , et al. Metformin inhibits androgen‐induced IGF‐IR up‐regulation in prostate cancer cells by disrupting membrane‐initiated androgen signaling. Endocrinology. 2014;155(4):1207–21. 10.1210/en.2013-1925 24437490 PMC3959597

[59] Shen M , Zhang Z , Ratnam M , Dou QP . The interplay of AMP‐activated protein kinase and androgen receptor in prostate cancer cells. J Cell Physiol. 2014;229(6):688–95. 10.1002/jcp.24494 24129850 PMC3947449

[60] Wang Y , Yao B , Wang Y , Zhang M , Fu S , Gao H , et al. Increased FoxM1 expression is a target for metformin in the suppression of EMT in prostate cancer. Int J Mol Med. 2014;33(6):1514–22. 10.3892/ijmm.2014.1707 24676803

[61] Zhang J , Shen C , Wang L , Ma Q , Xia P , Qi M , et al. Metformin inhibits epithelial‐mesenchymal transition in prostate cancer cells: Involvement of the tumor suppressor miR30a and its target gene SOX4. Biochem Biophys Res Commun. 2014;452(3):746–52. 10.1016/j.bbrc.2014.08.154 25201727

[62] Giannoni E , Taddei ML , Morandi A , Comito G , Calvani M , Bianchini F , et al. Targeting stromal‐induced pyruvate kinase M2 nuclear translocation impairs oxphos and prostate cancer metastatic spread. Oncotarget. 2015;6(27):24061–74. 10.18632/oncotarget.4448 26183399 PMC4695170

[63] Loubière C , Goiran T , Laurent K , Djabari Z , Tanti JF , Bost F . Metformin‐induced energy deficiency leads to the inhibition of lipogenesis in prostate cancer cells. Oncotarget. 2015;6(17):15652–61. 10.18632/oncotarget.3404 26002551 PMC4558177

[64] Chien SW , Kuo DY , Liao JM , Wang PS , Yu CH . Growth modulation of diabetic factors and antidiabetic drugs on prostate Cancer cell lines. Chin J Physiol. 2016;59(2):109–18. 10.4077/CJP.2016.BAE368 27080466

[65] Galdieri L , Gatla H , Vancurova I , Vancura A . Activation of AMP‐activated protein kinase by metformin induces protein acetylation in prostate and ovarian Cancer cells. J Biol Chem. 2016;291(48):25154–66. 10.1074/jbc.M116.742247 27733682 PMC5122782

[66] White‐Al Habeeb NM , Garcia J , Fleshner N , Bapat B . Metformin elicits antitumor effects and downregulates the histone methyltransferase multiple myeloma SET domain (MMSET) in prostate Cancer cells. Prostate. 2016;76(16):1507–18. 10.1002/pros.23235 27404348

[67] Imada K , Shiota M , Kuroiwa K , Sugimoto M , Abe T , Kohashi K , et al. FOXO3a expression regulated by ERK signaling is inversely correlated with Y‐box binding Protein‐1 expression in prostate Cancer. Prostate. 2017;77(2):145–53. 10.1002/pros.23254 27699813

[68] Tong D , Liu Q , Liu G , Xu J , Lan W , Jiang Y , et al. Metformin inhibits castration‐induced EMT in prostate cancer by repressing COX2/PGE2/STAT3 axis. Cancer Lett. 2017;389:23–32. 10.1016/j.canlet.2016.12.031 28043910

[69] Antognelli C , Cecchetti R , Riuzzi F , Peirce MJ , Talesa VN . Glyoxalase 1 sustains the metastatic phenotype of prostate cancer cells via EMT control. J Cell Mol Med. 2018;22(5):2865–83. 10.1111/jcmm.13581 29504694 PMC5908125

[70] Landim BC , de Jesus MM , Bosque BP , Zanon RG , da Silva CV , Góes RM , et al. Stimulating effect of palmitate and insulin on cell migration and proliferation in PNT1A and PC3 prostate cells: Counteracting role of metformin. Prostate. 2018;78(10):731–42. 10.1002/pros.23517 29635803

[71] Müller S , Versini A , Sindikubwabo F , Belthier G , Niyomchon S , Pannequin J , et al. Metformin reveals a mitochondrial copper addiction of mesenchymal cancer cells. PLoS ONE. 2018;13(11). 10.1371/journal.pone.0208213 PMC6219783 30399175

[72] Hou M , Venier N , Sugar L , Musquera M , Pollak M , Kiss A , et al. Protective effect of metformin in CD1 mice placed on a high carbohydrate‐high fat diet. Biochem Biophys Res Commun. 2010;397(3):537–42. 10.1016/j.bbrc.2010.05.152 20573602

[73] Pircher A , Zieher M , Eigentler A , Pichler R , Schäfer G , Fritz J , et al. Antidiabetic drugs influence molecular mechanisms in prostate cancer. Cancer Biol Ther. 2018;19(12):1153–61. 10.1080/15384047.2018.1491490 30067448 PMC6301819

[74] Nguyen MM , Martinez JA , Hsu CH , Sokoloff M , Krouse RS , Gibson BA , et al. Bioactivity and prostate tissue distribution of metformin in a preprostatectomy prostate cancer cohort. Eur J Cancer Prev. 2018;27(6):557–62. 10.1097/CEJ.0000000000000394 28692586 PMC5756696

[75] Hanahan D , Weinberg RA . Hallmarks of cancer: The next generation. Cell. 2011;44(5):646–74. 10.1016/j.cell.2011.02.013 21376230

[76] Graham GG , Punt J , Arora M , Day RO , Doogue MP , Duong JK , et al. Clinical pharmacokinetics of metformin. Clin Pharmacokinet. 2011;50(2):81–98. 10.2165/11534750-000000000-00000 21241070

[77] Williams GH , Stoeber K . The cell cycle and cancer. J Pathol. 2012;226(2):352–64. 10.1002/path.3022 21990031

[78] Banyard J , Chung I , Migliozzi M , Phan DT , Wilson AM , Zetter BR , et al. Identification of genes regulating migration and invasion using a new model of metastatic prostate cancer. BMC Cancer. 2014;14(1):387. 10.1186/1471-2407-14-387 24885350 PMC4046438

[79] Evans JMM , Donnelly LA , Emslie‐Smith AM , Alessi DR , Morris AD . Metformin and reduced risk of cancer in diabetic patients. BMJ. 2005;330(7503):1304–5. 10.1136/bmj.38415.708634.F7 15849206 PMC558205

[80] Baenke F , Peck B , Miess H , Schulze A . Hooked on fat: The role of lipid synthesis in cancer metabolism and tumour development. Dis Model Mech. 2013;6(6):1353–63. 10.1242/dmm.011338 24203995 PMC3820259

[81] Wu JD , Odman A , Higgins LM , Haugk K , Vessella R , Ludwig DL , et al. Effects of the human type I insulin‐like growth factor receptor antibody A12 on androgen‐dependent and androgen‐independent xenograft human prostate tumors. Clin Cancer Res. 2005;11(8):3065–74. 10.1158/1078-0432.CCR-04-1586 15837762

[82] Simpson A , Petnga W , Macaulay VM , Weyer‐Czernilofsky U , Bogenrieder T . Insulin‐like growth factor (IGF) pathway targeting in Cancer: Role of the IGF Axis and opportunities for future combination studies. Target Oncol. 2017;12(5):571–97. 10.1007/s11523-017-0514-5 28815409 PMC5610669

[83] Xia Y , Shen S , Verma IM . NF‐κB, an active player in human cancers. Cancer Immunol Res. 2014;2(9):823–30. 10.1158/2326-6066.CIR-14-0112 25187272 PMC4155602

[84] Collins KK . The diabetes‐Cancer link. Diabetes Spectrum. 2014;27(4):276–80. 10.2337/diaspect.27.4.276 25647050 PMC4231938

[85] Jurmeister S , Ramos‐Montoya A , Neal DE , Fryer LGD . Transcriptomic analysis reveals inhibition of androgen receptor activity by AMPK in prostate cancer cells. Oncotarget. 2014;5(11):3785–99. 10.18632/oncotarget.1997 25003216 PMC4116520

[86] Zhou J , Huang W , Tao R , Ibaragi S , Lan F , Ido Y , et al. Inactivation of AMPK alters gene expression and promotes growth of prostate cancer cells. Oncogene. 2009;28(18):1993–2002. 10.1038/onc.2009.63 19347029 PMC2679420

[87] Choudhury Y , Yang Z , Ahmad I , Nixon C , Salt IP , Leung HY . AMP‐activated protein kinase (AMPK) as a potential therapeutic target independent of PI3K/Akt signaling in prostate cancer. Onco Targets Ther. 2014;1:446–56. 10.18632/oncoscience.49 PMC4284621 25594043

[88] O'brien AJ , Villani LA , Broadfield LA , Houde VP , Galic S , Blandino G , et al. Salicylate activates AMPK and synergizes with metformin to reduce the survival of prostate and lung cancer cells ex vivo through inhibition of de novo lipogenesis. Biochem J. 2015;469(2):177–87. 10.1042/BJ20150122 25940306

[89] Jung S‐N , Park IJ , Kim MJ , Kang I , Choe W , Kim SS , et al. Down‐regulation of AMP‐activated protein kinase sensitizes DU145 carcinoma to Fas‐induced apoptosis via c‐FLIP degradation. Exp Cell Res. 2009;315(14):2433–41. 10.1016/j.yexcr.2009.05.018 19477172

[90] Chhipa RR , Wu Y , Mohler JL , Ip C . Survival advantage of AMPK activation to androgen‐independent prostate cancer cells during energy stress. Cell Signal. 2010;22(10):1554–61. 10.1016/j.cellsig.2010.05.024 20570728 PMC4712644

[91] Park HU , Suy S , Danner M , Dailey V , Zhang Y , Li H , et al. AMP‐activated protein kinase promotes human prostate cancer cell growth and survival. Mol Cancer Ther. 2009;8(4):733–41. 10.1158/1535-7163.MCT-08-0631 19372545 PMC2775041

[92] Carver BS , Chapinski C , Wongvipat J , Hieronymus H , Chen Y , Chandarlapaty S , et al. Reciprocal feedback regulation of PI3K and androgen receptor signaling in PTEN‐deficient prostate cancer. Cancer Cell. 2011;19(5):575–86. 10.1016/j.ccr.2011.04.008 21575859 PMC3142785

[93] Colquhoun AJ , Venier NA , Vandersluis AD , Besla R , Sugar LM , Kiss A , et al. Metformin enhances the antiproliferative and apoptotic effect of bicalutamide in prostate cancer. Prostate Cancer Prostatic Dis. 2012;15(4):346–52. 10.1038/pcan.2012.16 22614062

[94] Alghandour R , Ebrahim MA , Elshal AM , Ghobrial F , Elzaafarany M , Elbaiomy MA . Repurposing metformin as anticancer drug: Randomized controlled trial in advanced prostate cancer (MANSMED). Urol Oncol: Semin Orig Investi. 2021;39(831):e831–831.e810. 10.1016/j.urolonc.2021.05.020 34167872

[95] Tiwari R , Fleshner N . The role of metformin, statins and diet in men on active surveillance for prostate cancer. World J Urol. 2022;40(1):61–9. 10.1007/s00345-021-03858-4 34657209

[96] University Health Network , T. A Randomized, Double‐Blind, Placebo‐controlled trial of metformin in reducing progression among men on expectant Management for low Risk Prostate Cancer: The MAST (metformin active surveillance trial) Study (2021).

[97] Owen MR , Doran E , Halestrap AP . Evidence that metformin exerts its anti‐diabetic effects through inhibition of complex 1 of the mitochondrial respiratory chain. Biochem J. 2000;348(3):607–14. 10.1042/bj3480607 10839993 PMC1221104

[98] Iversen AB , Horsman MR , Jakobsen S , Jensen JB , Garm C , Jessen N , et al. Results from 11C‐metformin‐PET scans, tissue analysis and cellular drug‐sensitivity assays questions the view that biguanides affects tumor respiration directly. Sci Rep. 2017;7(1):9436. 10.1038/s41598-017-10010-z 28842630 PMC5573362

[99] Varghese S , Samuel SM , Varghese E , Kubatka P , Büsselberg D . High glucose represses the anti‐proliferative and pro‐apoptotic effect of metformin in triple negative breast Cancer cells. Biomolecules. 2019;9(1). 10.3390/biom9010016 PMC6359242 30626087

