# Supplementary material for: Systematic review of antitumour efficacy and mechanism of metformin activity in prostate cancer models
Source: BJUI Compass. 2022 Sep 30;4(1):44–58. doi: 10.1002/bco2.187 (PMC9766874; doi:10.1002/bco2.187)
Supplement: Supplementary file 2 — Table S4: Embase search strategy. Articles included from 2004 till 20 May 2020. [file BCO2-4-44-s002.docx]

**Supplementary Table 4: Embase search strategy.** Articles included from 2004 till 20 May 2020.

| Population | Intervention group | Outcome |
| --- | --- | --- |
| Anatomy   1. Exp prostate/ 2. (Prostate OR prostate gland OR prostat*).tw 3. OR/1-2   Pathology   1. (tumo?r* OR mass* OR adenocarcinoma OR growth* OR malignan* OR cancer* OR neoplas*).tw 2. (3 adj3 4). tw 3. Exp prostate neoplasms/ 4. OR/5-6 5. Exp metastasis 6. metastas*.tw 7. (7 adj3 8).tw 8. (7 adj3 9).tw 9. 7 or 10 or 11 | 1. Exp Metformin/ 2. metformin.tw 3. Exp Phenformin/ 4. phenformin.tw 5. OR/13-16 | Risk/outcome   1. (risk* or incidence* or prognosis* or outcome* or morbidity* or mortality*).tw   Decrease tumour growth   1. reduc* OR slow* OR decreas* OR inhibit* OR prevent*).mp 2. ((tumo?r OR neoplas* OR cell* OR cancer*) adj3 (proliferat* OR grow* OR replicat* OR progression)).mp 3. (19 ajd3 20).tw 4. Increas* OR enhance*.tw 5. (apoptosis* or cell death* or autophagy*).tw 6. (22 adj4 23).tw 7. (antineoplas* OR anticancer* OR antitumo?r* OR anti-cancer).tw 8. 21 or 24 or 25   Recurrence   1. Exp Recurrence/ 2. Exp Neoplasm Recurrence, Local/ 3. Exp Symptom Flare Up/ 4. Exp Neoplasm, Residual/ 5. Recurren*.mp 6. Relaps*.mp 7. Residual tumo?r.mp 8. Return*.mp 9. Reoccurren*.mp 10. Recrudescen*.mp 11. Reappearan*.mp 12. OR/27-37   No antiproliferative effect on PCa cells/tumour   1. Exp Treatment failure/ 2. Fail*.mp 3. OR/39-40   Metabolism   1. Exp Metabolomics/ 2. exp Metabolism/ 3. metabolomic*.tw 4. metabolism.tw 5. OR/42-45   Resistance to current therapy/sensitizes to current therapy   1. Exp pharmaceutical preparations/ 2. Androgen deprivation therapy.tw 3. exp drug therapy 4. radiation.tw 5. (drug* OR androgen deprivation therapy OR chemotherap* OR radiation* Or radiotherapy* OR therap* OR pharmacotherap*). tw 6. OR/47-51 7. Exp drug resistance/ 8. resistance.tw 9. OR/53-54 10. (51 adj5 55).tw 11. 18 or 26 or 38 or 41 or 46 or 56 or 52 |
| 1. 12 and 17 and 57🡪 1037 (many were supplements posted with different versions of abstract) 2. Limit 58 to (full text and English language) 🡪220. | | |
